# The clinical value and cost-effectiveness of treatments for patients with coronary artery disease

**DOI:** 10.1186/s13561-022-00401-y

**Published:** 2022-11-08

**Authors:** Huang Weiting, Alwin Zhang Yaoxian, Yeo Khung Keong, Shao Wei Lam, Lau Yee How, Anders Olof Sahlén, Ahmadreza Pourghaderi, Matthew Che, Chua Siang Jin Terrance, Nicholas Graves

**Affiliations:** 1 National Heart Centre, Singapore, Singapore; 2 Health Services Research Centre, SingHealth, Singapore, Singapore; 3 Duke NUS Medical School, 8 College Rd, 169857 Singapore, Singapore; 4 NUS, Singapore, Singapore

**Keywords:** Coronary artery bypass grafting, Percutaneous coronary intervention, Optimal medical therapy, Cost-effectiveness

## Abstract

**Background:**

The clinical value and cost-effectiveness of invasive treatments for patients with coronary artery disease is unclear. Invasive treatments such as coronary artery bypass grafting and percutaneous coronary intervention are frequently used as a starting treatment, yet they are much more costly than optimal medical therapy. While patients may transition into other treatments over time, the choices of starting treatments are likely important determinants of costs and health outcomes. The aim is to predict by how much costs and health outcomes will change from a decision to use different starting treatments for patients with coronary artery disease in an Asian setting.

**Methods:**

A cost-effectiveness study using a Markov model informed by data from Singapore General Hospital was done. All patients with initial presentations of stable coronary disease and no acute coronary syndromes who received medical treatments and interventional therapies were included. We compare existing practice, where the starting treatment can be medical therapy or stent percutaneous coronary interventions or coronary artery bypass grafting, with alternate starting treatment strategies.

**Results:**

When compared to ‘existing practice’ a policy of starting 14% of patients with coronary artery bypass grafting and 86% with optimal medical therapy showed savings of $1,743 per patient and 0.23 additional quality adjusted life years. A change to policy nationwide would save $10 million and generate 1,380 quality adjusted life years.

**Conclusions:**

Increasing coronary artery bypass grafting and use of medical therapy in the setting of coronary artery disease is likely to saves costs and improve health outcomes. A definitive study to address the question we investigate would be very difficult to undertake and so using existing data to model the expected outcomes is a useful tool. There are likely to be large and complex barriers to the implementation of any policy change based on the findings of this study.

## Introduction

There is debate over the clinical value and cost-effectiveness of invasive treatments for patients with triple vessel coronary artery disease [1, 2]. While coronary artery bypass grafting (CABG) is recommended for triple vessel disease, especially in the presence of diabetes mellitus and severe left ventricular systolic dysfunction [3], many patients choose multi-vessel percutaneous coronary intervention (PCI) over CABG as their starting treatment option [4, 5]. When patients decline guideline-recommended CABG, less aggressive and temporal management of some patients with medical therapy could be a sensible option. Studies comparing optimal medical therapy and PCI in stable coronary artery disease did not support starting with a strategy of PCI, as it did not improve major cardiovascular outcomes compared to optimal medical therapy alone [6, 7]. However, patients can transition to PCI over time when optimal medical therapy fail to adequately control angina. While patients may transition into other treatments over time, the choices of starting treatments are likely important determinants of costs and health outcomes [8].

Prior clinical trials have consistently demonstrated CABG to be an economically attractive option compared to percutaneous coronary intervention [9] [10]. There are few real world population studies, and they were mainly performed in Western populations [11]. The cost of care in controlled clinical trials are likely differ from real world services [12]. Moreover, most studies also only compare 2 arms, CABG versus PCI, excluding the optimal medical therapy arm which is increasingly important in the treatment of ischemic heart disease [13].

The use of the PCI as a starting treatment, especially in minimally symptomatic patients when they have declined surgery, needs to be considered against the context of healthcare growing costs. In Singapore costs increased by 300% between 2000 and 2016 [14]. High cost and frequently used acute services such as cardiology should to be at the forefront of the cost-effectiveness agenda, given increasing demand for scarce resources.

Our study will include CABG, PCI and optimal medical therapy as starting treatments. This will cover the entire spectrum of clinical care for coronary artery disease and to provide a holistic view from a cost perspective in an Asian population. The purpose of the analysis is to summarise data on the competing treatment strategies for coronary artery disease from a cost-effectiveness standpoint. We will identify the types and timings of treatments provided for all patients with stable coronary artery disease (CAD) under current practices in Singapore. We expect some patients to have invasive treatments as their starting treatment, and others to start with medical therapies. We will summarise the longitudinal costs and health outcomes for all patients, grouped by their starting intervention. We then model naïve changes to practice where we assume 100% of all individuals receive CABG, medical management or stent PCI as their starting treatment. This will provide insights into how costs and health outcomes are determined by the choice of starting treatment. We then model a more realistic blended policy option, based on clinical judgments and existing evidence, that reveal changes to total costs and health benefits; and we examine the trade-offs. We address, to some extent, the knowledge gap of how costs and outcomes vary among competing treatment options for CAD.

The aim is to predict, with uncertainty, by how much costs and health outcomes will change from a decision to use different starting treatments for patients with CAD in a Singapore setting. We will use the cost-effectiveness paradigm to shed some light on the question of how the economic performance of cardiology services might be improved.

## Methods

### Data, setting and patients

The data used for this study were harvested from patient medical records for 2011 to 2014 eHINTs [15]. This enterprise data repository includes administration, cost and clinical information for patients who had visited either the Singapore General Hospital or the National Heart Centre, which is a separate national referral centre for cardiovascular diseases. We include patients with presentations of stable coronary disease and no acute coronary syndromes who received these treatments: CABG; any type of stent PCI; or, optimal medical therapy (OMT) medication which included at least antiplatelet therapy and statin drugs.

### Model structure and assumptions

A Markov model was developed using R 4.0.1 software [16] with the package ‘heemod’ [17] to evaluate the health services costs and health outcomes associated with the competing treatment pathways seen in the patient data. Markov models are used for decision modelling in cost-effectiveness analysis as they accommodate competing clinical pathways and time dependency and enable simulations to show uncertainty in the data. They also allow the comparison of policy options and can show multiple outcomes [18]. The cycle length is 6 months and the total number of cycles were determined through domain knowledge on CAD and estimation of the treatment trajectory. The perspective of the analysis was one that included health services costs and a discount rate of 3% was applied to future costs and health benefits [19]. The threshold for cost-effectiveness was selected based on the latest per capita GDP in Singapore which is USD 59,800 or approximately SGD $80,000 [17]. This amount is based on an assumption that one year of perfect quality life is worth the per capita gross domestic product [20].

### Comparisons between competing treatment pathways

**Fig. 1:**
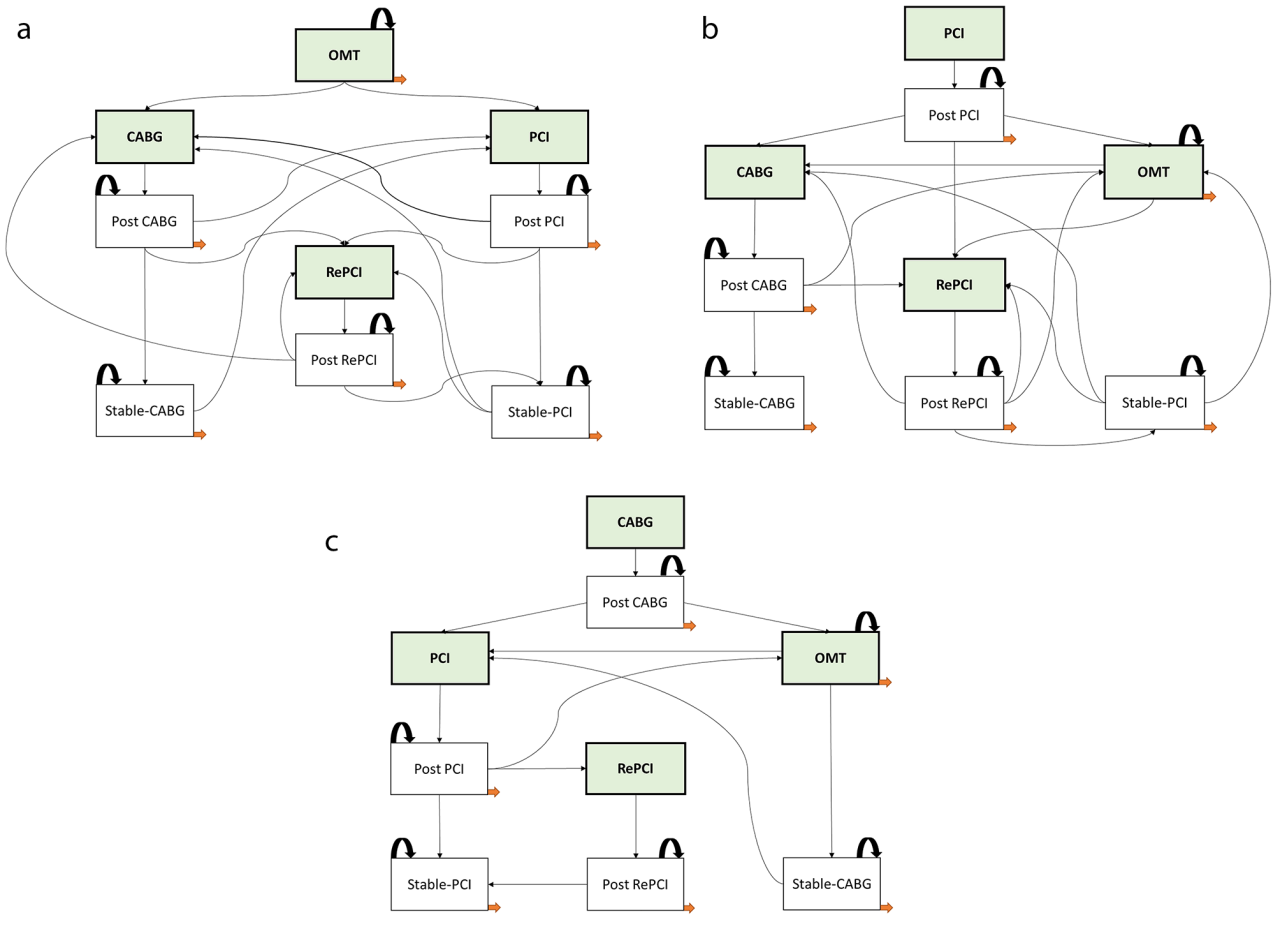
(**a**) State transition diagram for those starting with OMT. (**b**) State transition diagram for those starting with PCI. (**c**) State transition diagram for those starting with CABG.

The Markov model diagram in Fig. 1 is used to show the treatment journeys for patients whose starting treatment was optimal medical therapy (OMT), stent percutaneous coronary interventions (PCI) or coronary artery bypass grafting (CABG). All patients face probabilities of treatments and outcome states including death and stable disease. Post-surgery states for procedures requiring surgical interventions such as PCI, repeat PCI (RePCI) and CABG were included to account for long-term costs incurred. The stable PCI or CABG states are for patients with a large probability of maintaining outcomes, rather than transitioning to any other states.

Current practice was defined by a cohort with the following distribution among starting treatment: 81.9% OMT, 13.5% PCI and 4.6% CABG. This was the baseline for comparison with four competing options: 100% patients starting with CABG; 100% patients starting with OMT; 100% patients starting with PCI; and, a recommended policy of 86% starting OMT and 14% CABG.

The first three options of 100% allocation to a starting treatment are naïve but somewhat useful to illustrate the impact of different decisions on costs and health benefits. The more informative ‘Recommended Policy’ is based on the existing knowledge that 14% of annual patient visits who were diagnosed with CAD and had either LM disease or triple vessel disease, with the optimal therapy of choice being coronary artery bypass grafting surgery. Based on existing literature and expert opinion we assume zero patients receive stent PCI as the starting treatment.

### Transition probabilities

Most of the transition probabilities were estimated from the patient medical records for 2011 to 2014. The proportion of the total number of patients in a particular state who transitioned into a different state in a given cycle was calculated. The probability of transitions to stable PCI and stable CABG were derived from published data [21], with 64 out of 70 patients who had bypass surgery and 43 out of 72 who had angioplasty (PCI) found to be free from acute myocardial infarction, presence of refractory angina, or death after a 5-year follow-up. These probabilities were rescaled from 5 years to 6 months making use of the cumulative incidence proportion equation which factors an exponential decay across time [22]. This rescaling can be done using specific functions via the ‘heemod’ library on R. The values for the transition probabilities may be found in the appendix section. Beta and Dirichlet distributions were used to show the uncertainty in these parameters.

### Costs & health utilities

The parameter estimates for costs and health utilities for each of the model states are shown in Table 1, including the distributions for modelling uncertainties. The cost of surgical procedures for PCI and CABG are estimated based on the ‘Fee Benchmarks and Bill Amount Information’ provided by the Ministry of Health [23]. This information contains the historical total transacted bill sizes for various surgical procedures in public hospitals based on the different ward classes as well as private hospitals. The average costs were computed to reflect the full costs of supply, incurred by the hospital. The cost of pharmaceuticals for medical therapy were obtained directly from the patient’s billing data, and the average cost per patient was computed based on medication costs incurred over a 6-month period. As the OMT costs was expected to be different in patients who had undergone a procedure as compared to those who did not, the OMT costs for three different groups was computed: costs for patients who had a history of CAD but had not undergone any interventional procedure; costs for patients who undergone a stent procedure; costs for patients who undergone a bypass graft procedure.

**Table 1:** Parameter Estimates and Distributions for Costs and Health Utilities

Parameters	Estimate, Mean (SD)	Distribution
Average Costs		
OMT, without intervention	$129 (36)	Gamma (129.86, 36.06)
OMT, after stent procedure	$147 (14)	Gamma (147.68, 14.29)
OMT, after bypass grafting	$96 (30)	Gamma (96.65, 30.03)
PCI	$26,261 (4908)	Gamma (26261.18, 4908.53)
CABG	$35,756 (4419)	Gamma (35756.64, 4419.17)
Heath Utility		
OMT	0.69 (0.12)	Beta (10.43, 4.63)
PCI	0.72 (0.22)	Beta (2.37, 0.90)
Re-PCI	0.70 (0.16)	Beta (165.01, 37.04)
Stable PCI	0.87 (0.03)	Beta (4.93, 2.13)
CABG	0.82 (0.03)	Beta (101.27, 0.031)
Stable CABG	0.84 (0.04)	Beta (79.04, 15.01)

The estimates for health utility weights were obtained from a systematic review consisting of quality-of-life measurements by EQ-5D for various treatment states at different treatment time points for patients with CAD, where a random effects model was used to consolidate the values from different studies [24]. The values for each treatment or health state were taken at 6 months, whereas the stable states after each invasive intervention were taken over a longer term at 36 months. Repeated PCI procedures were assigned the values for acute re-hospitalization utility weights since patients who have multiple repeated surgeries are assumed to have lower health utilities.

### Model evaluation and uncertainty

A cohort of 10,000 patients was used for the Markov model running with 20 cycles over 10 years. To account for events and transitions that can occur at any point during the cycle, a half-cycle correction was included. A 3% discount rate was used for future costs and health outcomes in concordance with recommendations of the Panel on Cost-effectiveness in Health and Medicine [25]. An average increase in death rate by 0.01 every 10 cycles was also incorporated into the model on top of the death probabilities computed within each of the states in Fig. 1 using data for mortality rates according to World Health Organization (WHO) by country, age, and gender [26].

Probabilistic sensitivity analysis was used to include parameter uncertainty for the estimates of costs and health utilities based on the sampling distributions. Costs were assumed to follow a gamma distribution as they were right skewed non-negative values where a larger portion of patients had lower expenditures and a smaller fraction incur higher expenditures. Health utilities were assumed to follow a beta distribution as all values were between 0 and 1. Distributions were generated around the baseline estimates for costs and health utilities and were sampled 1000 times.

The primary outcomes reported are the mean incremental cost per QALY gained for each comparison and a linear version of this information expressed as net monetary benefits (NMB). NMB is a summary statistic that represents the value of an intervention in monetary terms when a willingness-to-pay (WTP) threshold for a unit of benefit (or QALY) is known. It is calculated by first assuming a WTP threshold and converting QALYs into the common metric of dollars and then subtracting the cost associated with each treatment strategy to result in the net benefit of each strategy expressed in the monetary units [27].

### Summary of patient characteristics

To summarise the characteristics of the patients by the different starting groups, continuous variables were expressed as a mean value and categories were expressed in terms of counts and proportions. A 95% confidence interval was used to represent the range of intervals which the values could occur, where a normal distribution was used for age and Poisson distribution for counts. Institutional Review Boards approvals are waived as all data are pre-existing, retrospective and anonymized.

## Results

### Population characteristics

The characteristics of the population who had visited either Singapore General Hospital or the National Heart Centre for treatment of CAD between 2011 and 2014 are shown in Table 2.

**Table 2:** Baseline characteristics of the population who received treatment for CAD

	OMT (n = 19,467)	95% CI	PCI (n = 3205)	95% CI	CABG (n = 1102)	95% CI
Age, mean	62.96	62.80–63.12	58.85	58.47–59.23	60.84	60.30–61.39
Male	13,521 (69%)	13,294–13,751	2,650 (83%)	2550–2753	911 (83%)	853–972
Female	5,913 (30%)	5763–6066	546 (17%)	501–594	189 (17%)	163–218
Chinese	13,853 (71%)	13,623–14,086	2005 (63%)	1918–2095	712 (65%)	661–766
Malay	2,266 (12%)	2173–2361	475 (15%)	433–520	161 (15%)	137–188
Indian	2,498 (13%)	2401–2598	424 (13%)	385–466	115 (10%)	95–138
Others	850 (4%)	794–909	301 (9%)	268–337	114 (10%)	94–137
Diag LM	255 (1%)	225–288	67 (2%)	52–85	22 (2%)	14–33
Diag TVD	3,780 (19%)	3660–3902	588 (18%)	438–525	480 (44%)	438–525
LM & TVD	1,935 (10%)	1850–2023	143 (4%)	431–518	473 (43%)	431–518
Diabetes	8,453 (43%)	8274–8635	1185 (37%)	1119–1254	504 (46%)	461–550
Hypertension	14,456 (74%)	14,221–14,694	2157 (67%)	2067–2250	838 (76%)	782–897
Dyslipidemia	15,251 (78%)	15,010–15,495	2430 (76%)	2334–2529	911 (83%)	853–972
LVEF ≤ 40%	3,290 (17%)	3179–3404	441 (14%)	401–484	215 (20%)	187–246

Notes: LVEF ≤ 40% = left ventricular dysfunction; TVD = triple vessel disease

Among the 23,774 study patients, 19,467 (81.9%) had OMT as their starting treatment. Among those who started with an intervention 13.5% underwent PCI as compared to 4.6% having a CABG. Demographics in terms of age, gender, race and risk factors were quite similar. In terms of severity of coronary anatomy, the groups were largely similar except in the most severe left main and triple vessel disease, which had lower prevalence in both the PCI and OMT arm, suggesting that the most severe disease were still preferentially treated with CABG. Although evidence strongly suggest for CABG in patients with diabetes and left ventricular dysfunction less than 40% [28], there was no significant trend toward choosing CABG as the choice treatment option amongst these groups of patients.

### Costs effectiveness outcomes

**Fig. 2:**
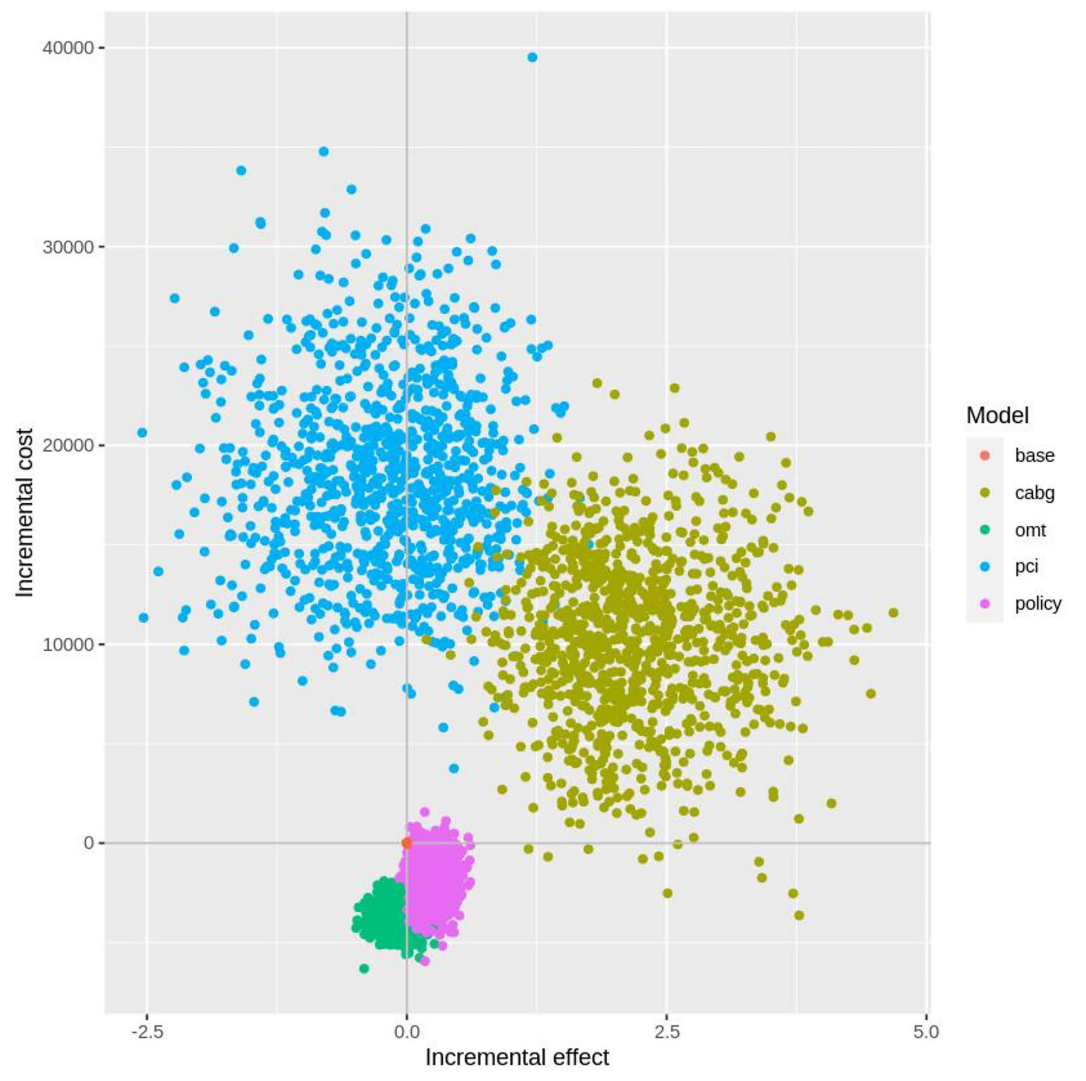
Incremental cost-effectiveness plane, with 1000 samples plotted for each option

The joint distributions of the incremental change to costs and QALYs from all competing options as compared to current practices are shown in Fig. 2. The current practice is at the origin of the axes, labelled ‘base’.

The changes in costs, QALYs and NMB are shown in Table 3.

**Table 3:** Changes to Costs, QALYs and NMB as compared to current practice

	Change to Costs (95% UI)	Change to QALYs (95% UI)	NMB (95% UI)
100% patients starting with CABG	$10,040 (9766–10,314)	2.19 (2.14–2.23)	$164,957 (161,241–168,673)
100% patients starting with OMT	-$3661 (-3700–3622)	-0.09 (-0.1–0.08)	-$13,630 (-17,270–9990)
100% patients starting with PCI	$18,789 (18,484–19,093)	-0.21 (-0.26–0.17)	-$36,080 (-39,715–32,445)
Recommended Policy	-$1743 (-1808 -1678)	0.23 (0.22–0.24)	$20,240 (19,637–20,844)

The results show that the ‘100% patients starting with PCI’ option was higher cost, with 100% certainty, than current practice and also reveal a 58.8% probability it provided worse health outcomes. It was certain the ‘100% patients starting with CABG’ option will increase costs and increase health outcomes compared to current practice. The ‘100% patients starting with medical management’ option showed evidence of saving costs without significantly improving patients’ outcomes from baseline. These naïve strategies are not useful for policy making since it is not realistic to send the entire population to start with a specific treatment. Rather the recommended policy of 86% starting OMT and 14% CABG was evaluated and yielded a favorable outcome as compared to current practice.

On average there would be expected cost savings of $7538 per QALY gained from adoption of the ‘Recommended Policy’ as compared to current practice. There is a 95.4% probability that adoption will save costs, a 99.1% probability it will improve health outcomes and a 99.7% probability it will be cost effective against the chosen threshold of $80,000 per QALY gained.

## Discussion

This research suggests that when compared to existing practice a decision is to adopt the ‘Recommended Policy’ of 14% CABG and 86% OMT is cost saving and generates extra health benefits. Patients may proceed to invasive interventions, should OMT not be sufficient to control angina, and CABG is the preferred option over PCI. This is quite different from existing practice where 13.5% of patients start with PCI. Adoption of the recommended policy implies a large expansion of CABG and a modest increase of OMT as starting treatments. If this were implemented than there would be expected savings of $1743 per patient with CAD and a gain in QALYs of 0.23. Assuming 6,000 patients are treated per year this would save over $10 million in costs and generate a gain of 1,380 QALYs, with health these benefits valued at $80,000 each the economic value of the aggregate health benefit is close to $110 M per year. Our findings add weight to a recent systematic review [2] that reported evidence from five cost effectiveness studies about treatments for stable coronary artery disease.

Making changes to policy requires an understanding of the objectives of multiple relevant stakeholders. For those who manage health services, a major consideration should be to improve value for money from spending on health services, and this analysis provides some clear signals. There are however barriers to the use of economic evaluation studies by decision-makers[24]. Merlo et al. [29] adapted an existing framework [30] to organise the barriers into ‘Accessibility’ and ‘Acceptability’ issues. The former refers to the ability of decision-makers to interpret and use economic evidence, while the latter speaks to the complexity and timeliness of economic evaluations and factors such as scientific rigor, applicability to the organisations making the decision and ethical considerations. One might expect that managing the preferences of interventional cardiologists, who are trained and remunerated for providing PCI stent, and patients, who may believe the treatments are beneficial are examples of large and important barriers.

Our study also supports the conclusions of existing clinical trials, but from a cost-effectiveness point of view. From the clinical perspective, studies such as the ISCHEMIA trial, where 45% of patients had triple vessel disease, routine invasive management with CABG or PCI did not reduce major adverse cardiovascular outcomes. Our analysis focused on the initial strategy of treatment when diagnosis is made, rather than the final treatment decision. A patient who continues to have activity limiting angina should proceed onto revascularization therapy if optimal medical therapy fails to control symptoms. In the ISCHEMIA trial, approximately 20% of patients in the medical therapy arm went onto revascularization. The authors also emphasize that in the setting of high-risk anatomy or high risk features, such as severe left main coronary artery disease and severe left ventricular systolic dysfunction, there is greater clinical urgency for revascularization with preferably CABG, as recommended by clinical guidelines.

An important caveat of our analysis is that we assume the patients in the three starting treatment groups are similar. The groups of patients studied are largely similar in age, demographics, race and risk factors. The obvious difference in treatment options is for the group with the most severe disease of left main and triple vessel disease, 10.7% of study population, where CABG was the preferred choice. A definitive study to address the question we investigate would be very difficult to undertake. A randomized trial among CABG, PCI and OMT for the purpose of studying cost effectiveness would be challenging for research integrity and review, when clinical evidence points towards CABG as the preferred revascularization option. Using our existing and rich data to model the impact of changing clinical practices is the next best available tool [31]. The authors also acknowledge that the decision for PCI is also largely patient driven, hence with this knowledge, physicians should spend more time convincing patients for CABG should they agree on intervention or remain symptomatic after OMT, from both clinical and cost perspective. Another limitation to this study is that the data used are almost 10 years old. There will have been change to treatments and the features of the patients, from aging and disease patterns that may affect the reliability of findings. Ideally more recent data will be interrogated to validate these results.

While the community should continue to examine information about improving the cost-effectiveness of cardiology services, it might be that the next research effort is targeted at the implementation issues. It is difficult to get new evidence about alternate ways of providing services translated widely into healthcare settings [32]. There are information, human and bureaucratic barriers at many of the steps [33]. Implementation science is an emerging and important discipline that proposes theory and evidence based methods and strategies to hasten the adoption of new evidence [30, 34]

## Data Availability

The data underlying this article cannot be shared publicly due to a policy that states routinely collected data are not made available to the public by SingHealth.

## Appendix Table A1: Transition Probabilities of OMT, PCI and CABG

**Table:**

OMT Start State											
	Next State										
Current State		OMT	CABG	Post CABG	Stable CABG	PCI	Post PCI	Re-PCI	Post Re-PCI	Stable PCI	Death
	OMT	C	0.03	0	0	0.061	0	0	0	0	0.041 + mr
	CABG	0	0	1	0	0	0	0	0	0	0
	PostCABG	0	0	C	0.218	0.025	0	0	0	0	0.05 + mr
	StableCABG	0	0	0	C	0	0	0	0	0	0.051 + mr
	PCI	0	0	0	0	0	1	0	0	0	0
	PostPCI	0	0.007	0	0	0	C	0.029	0	0.087	0.073 + mr
	RePCI	0	0	0	0	0	0	0	1	0	0
	PostRePCI	0	0	0	0	0	0	0.057	C	0.087	0.092 + mr
	StablePCI	0	0	0	0	0	0	0.01	0	C	0.005 + mr
	Death	0	0	0	0	0	0	0	0	0	1
PCI Start State											
	Next State										
Current State		OMT	CABG	Post CABG	Stable CABG	PCI	Post PCI	Re-PCI	Post Re-PCI	Stable PCI	Death
	OMT	C	0.004	0	0	0	0	0.129	0	0.087	0.029 + mr
	CABG	0	0	1	0	0	0	0	0	0	0
	PostCABG	0.25	0	C	0.218	0	0	0	0	0	0
	StableCABG	0	0	0	C	0	0	0	0	0	0.051 + mr
	PCI	0	0	0	0	0	1	0	0	0	0
	PostPCI	0.845	0.003	0	0	0	C	0.002	0	0	0.038 + mr
	RePCI	0	0	0	0	0	0	0	1	0	0
	PostRePCI	0.012	0.012	0	0	0	0	0.049	C	0.087	0.024 + mr
	StablePCI	0	0	0	0	0	0	0.047	0	C	0.256 + mr
	Death	0	0	0	0	0	0	0	0	0	1
CABG Start State											
	Next State										
Current State		OMT	CABG	Post CABG	Stable CABG	PCI	Post PCI	Re-PCI	Post Re-PCI	Stable PCI	Death
	OMT	C	0	0	0.218	0.028	0	0	0	0	0.022 + mr
	CABG	0	0	1	0	0	0	0	0	0	0
	PostCABG	0.844	0	C	0	0.009	0	0	0	0	0.033 + mr
	StableCABG	0	0	0	C	0	0	0	0	0	0.015 + mr
	PCI	0	0	0	0	0	1	0	0	0	0
	PostPCI	0.25	0	0	0	0	C	0.125	0	0.087	0
	RePCI	0	0	0	0	0	0	0	1	0	0
	PostRePCI	0	0	0	0	0	0	0	C	0.087	0.092 + mr
	StablePCI	0	0	0	0	0	0	0	0	C	0.005 + mr
	Death	0	0	0	0	0	0	0	0	0	1

Note: The abbreviations **mr** represents the mortality rate which increases by 1% every five years according to the data provided by WHO. **C** represents the complementary probability of all other row probabilities
